# Polymorphous low-grade adenocarcinoma of the tongue: a case report

**DOI:** 10.1186/1752-1947-3-9313

**Published:** 2009-12-02

**Authors:** Ruchi Gupta, Kirti Gupta, Rijuneeta Gupta

**Affiliations:** 1 Department of Histopathology and Department of Otolaryngology and Head and Neck Surgery, Postgraduate Institute of Medical Education and Research, Chandigarh, India

## Abstract

**Introduction:**

Polymorphous low-grade adenocarcinoma is a distinct neoplasm of the salivary gland composed of luminal and non-luminal tumor cells admixed in varying proportions. Its resemblance to lobular carcinoma of the breast had led to its earlier nomenclature of 'terminal duct carcinoma'. Most patients present with an asymptomatic mass in the hard palate. In rare cases, the mass can also occur in the tongue. We report an unusual case of polymorphous low-grade adenocarcinoma at the base of tongue.

**Case presentation:**

A 47-year-old Asian Caucasian woman presented with a painless swelling at the right lateral border of the tongue with an intact overlying mucosa. There were no other associated complaints. The lesion was excised and subjected to histopathological examination that revealed an interesting and unusual morphology of polymorphous low-grade adenocarcinoma.

**Conclusion:**

Polymorphous low-grade adenocarcinoma is a well-defined entity in the minor salivary glands. Its occurrence in the tongue is rare with very few cases reported in the literature. It is a malignant neoplasm with low aggressiveness and it is thus important to identify and treat it accordingly.

## Introduction

Polymorphous low-grade adenocarcinoma (PLGA) is a malignant neoplasm with a low level of aggressiveness that occurs almost exclusively in the minor salivary glands, primarily those in the palate. We report a case of PLGA that arose at the base of the tongue in a 47-year-old woman. The tumor was resected through the oral cavity with wide margins. The patient recovered and remained disease-free at follow-up. This case shows that PLGA, which has a variable morphologic appearance, can occur at sites other than the salivary glands.

## Case presentation

A 47-year-old Asian Caucasian woman presented with a painless swelling over the right lateral border of her tongue that had gradually increased over the four months prior to presentation. It had an insidious onset and progressively increased in size. The patient had no history of discharge, bleeding or ulceration over the swelling. On examination, the swelling was 3 × 2 cm in size and was located along the lateral border at the junction of the anterior 1/3rd and posterior 2/3rd. It was firm in consistency and well circumscribed with all the margins felt clearly. The patient had no restriction in the movement of her tongue. There was no significant peripheral lymphadenopathy. The lesion was excised and sent for histopathological examination.

**Figure 1:**
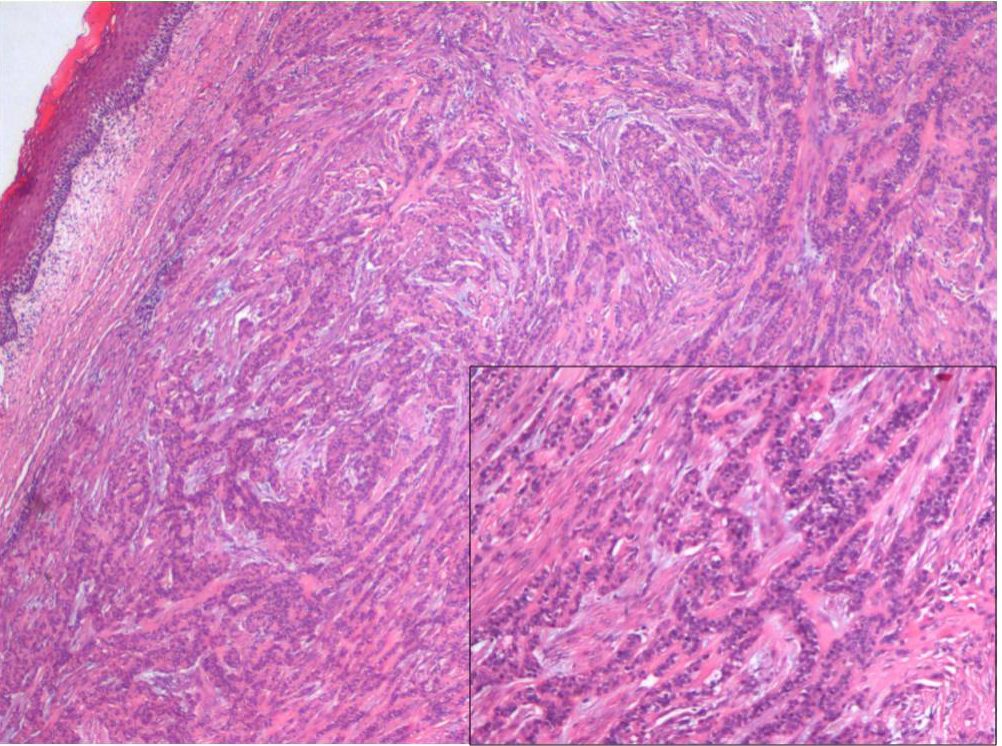
**Polymorphous low-grade adenocarcinoma located just beneath the mucosal stratified squamous epithelium of the tongue** (original magnification ×40, Hematoxylin and Eosin stain). Inset highlights the low cuboidal to oval cells arranged in cords and tubules embedded in a fibrous and/or hyalinized stroma (original magnification ×200, Hematoxylin and Eosin stain).

**Figure 2:**
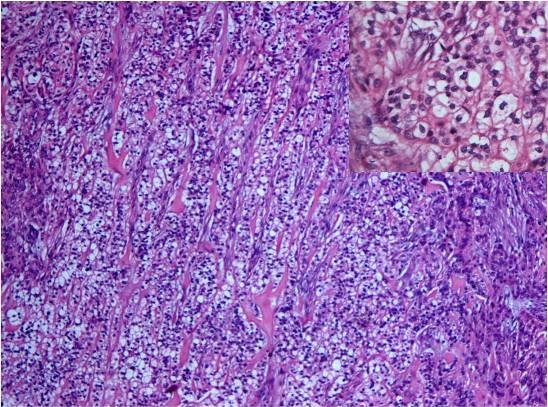
**Sheets of tumor cells with moderate amount of eosinophilic to clear cytoplasm, with finely dispersed granular chromatin (inset)** (original magnification ×200, Hematoxylin and Eosin stain, inset ×400).

**Figure 3:**
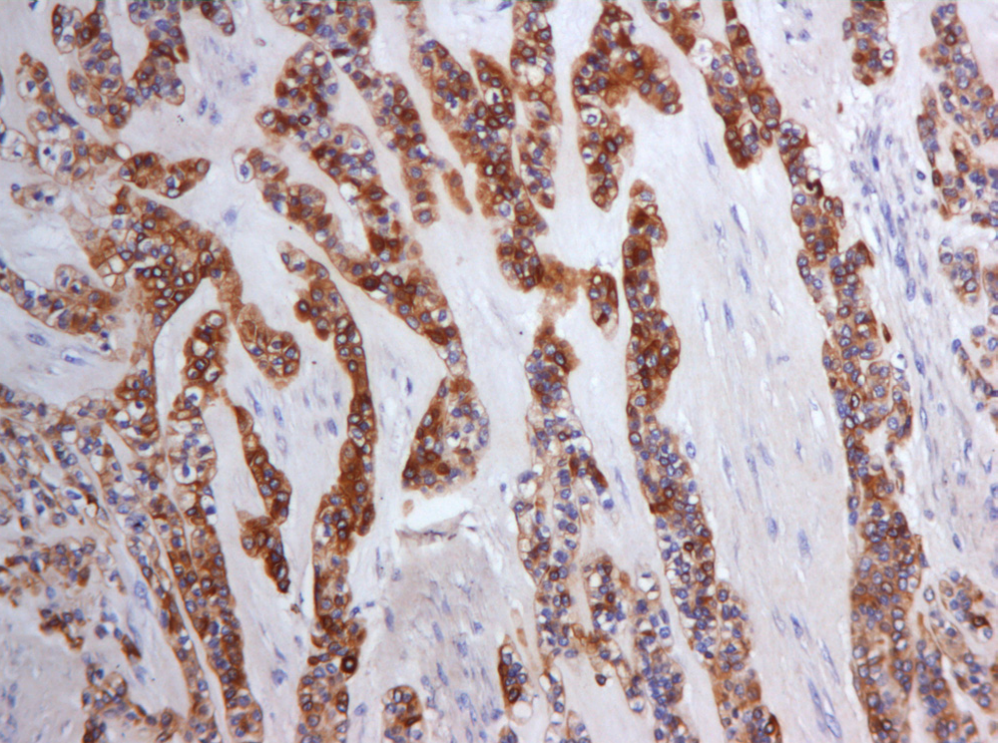
**Tumor cells show strong cytoplasmic positivity for cytokeratin** (original magnification ×400, immunoperoxidase stain).

On gross examination, it was discovered to be well-circumscribed lesion, 2 × 3 cm in size, firm in consistency and with a gray-white cut surface. Histological examination showed a relatively well-circumscribed tumor with focally infiltrative margins. The tumor cells were arranged in varied patterns: tubular, papillary, cords, and also in sheets (Figure 1). The tumor cells were monomorphic in appearance, round to oval with bland nuclear chromatin (Figure 1), and had a moderate amount of eosinophilic to clear cytoplasm (Figure 2). A small amount of intervening hyalinized stroma could be appreciated. The peripheral invasive component showed an 'Indian file' pattern of arrangement. Immunostain for cytokeratin was positive (Figure 3), while smooth muscle antigen (SMA) showed negative immunoreactivity.

## Discussion

The term polymorphous low-grade adenocarcinoma was first used in 1984 by Evans and Batsakis to describe a tumor of the salivary glands that had a variety of architectural patterns associated with cytologic uniformity as its primary histologic characteristic [1]. The most common sites of this tumor are the minor salivary glands in the palate, followed by buccal mucosa, lip, retromolar triangle, and the cheek [2]. In very rare cases, the tumor also occurs in the tongue [3-5].

PLGA had been previously referred to as terminal duct carcinoma in view of its probable origin in the ductal system of the salivary glands [2]. Similar to terminal duct carcinoma, PLGA is formed by luminal epithelial, myoepithelial, and basal epithelial cells [5]. Immunohistochemistry has as such no apparent diagnostic value in identifying this tumor. The tumor in our patient had positivity for pan-cytokeratin and a focal positivity for S-100 as has been described in the literature.

Because of its morphologic pleomorphism, PLGA has often been misdiagnosed as pleomorphic adenoma or adenoid cystic carcinoma (ACC) [6]. However, PLGA differs from pleomorphic adenoma because it is characterized by infiltrative margins and an absence of chondromyxoid stroma [6]. The primary difference between PLGA and ACC is based on both cytologic and histologic characteristics. Cell cytoplasm in PLGA is eosinophilic with rounded nuclear borders, while the cells in ACC are more basaloid with angled and hyperchromatic nuclei. It is important to distinguish ACC from PLGA because the former is associated with low long-term survival rates. PLGA is a low-grade malignancy, and its biologic behavior is apparently not influenced by the different morphologic and cell differentiation patterns that it may exhibit [7]. The only exception to this behavior is seen with tumors that have a predominantly papilliferous arrangement; these tumors are more aggressive and would be better classified as papillary cystadenocarcinomas [8].

## Conclusion

PLGA is an unusual tumor to occur at the base of the tongue. It is a low-grade aggressive neoplasm and it is important to recognize and distinguish it from other benign tumors known to occur at this site. The possibility of PLGA must be considered in cases of oral cavity tumors, such as the tongue.

## Abbreviations

ACC: adenoid cystic carcinoma; PLGA: polymorphous low-grade adenocarcinoma; SMA: smooth muscle antigen

## Consent

Written informed consent was obtained from the patient for publication of this case report and any accompanying images. A copy of the written consent is available for review by the Editor-in-Chief of this journal.

## Competing interests

The authors declare that they have no competing interests.

## Authors' contributions

KG and RG performed the histological examination of the tumor. They were also major contributors in writing the manuscript. GR analyzed and interpreted the patient clinical data and also carried out the excision of the lesion. All authors read and approved the final manuscript.
